# Association between neighbourhood cohesion and physical activity trajectories during the COVID-19 pandemic using data from *Understanding Society*: The UK Household Longitudinal Study & COVID-19 sub-study

**DOI:** 10.1016/j.pmedr.2023.102392

**Published:** 2023-08-29

**Authors:** Verity Hailey, Mikaela Bloomberg, Mark Hamer, Abi Fisher

**Affiliations:** a Department of Behavioural Science and Health, University College London, London, UK; b Institute Sport Exercise & Health, Division Surgery & Interventional Science, University College London, London, UK

**Keywords:** Neighbourhood cohesion, Physical activity, Lockdown, COVID-19, Social environment

## Abstract

Neighbourhood cohesion is increasingly recognised as a key determinant of health and health-related behaviours. Positive association between social support and physical activity have been demonstrated on an interpersonal level, there is less evidence at group-level. This study aimed to examine the association between neighbourhood cohesion and physical activity trajectories during the COVID-19 pandemic. Hypothesizing that higher neighbourhood cohesion was a protective factor against reduced physical activity during the pandemic. Data from *Understand Society* (wave 9, Jan 2017–Dec 2019), and the COVID-19 sub-study (waves 1, 5, 7) was used. Participants (N = 14,475) had baseline data and at least one Covid physical activity measure. We used linear mixed models with a random intercept and slope at the individual level and an unstructured correlation matrix to examine the association between neighbourhood cohesion and physical activity during the follow-up period. We found a significant reduction in physical activity (-441 MET-min/wk, (CI 374.51 – 507.65, p < 0.001) through the COVID-19 pandemic, and that higher neighbourhood cohesion was related to higher physical activity after control for covariates. There was a significant difference between neighbourhood cohesion categories and change seen in PA during the 39-month follow-up period (difference in change between lowest and highest neighbourhood cohesion categories = 373 MET-min/wk, p = 0.036), higher neighbourhood cohesion had a protective effect. Strong relationships between public health and urban planning sectors are needed to build communities with structures in place to support a sense of community, social interaction and attraction to the neighbourhood. This will help long-term neighbourhood cohesion and support increased physical activity.

## 1 Background

During the COVID-19 pandemic people spent more time in their neighbourhoods due to lockdowns, travel restrictions and working from home. As the pandemic recedes many people continue to work from home, and hybrid working has become more popular. Consequently, the neighbourhoods we live in becomes more important as we spend more time living and working in them. Most health behaviours are socially patterned with both the social and physical environment impacting behaviours (McNeill et al., 2006). Neighbourhood factors are increasingly recognised as determinants of health and of health behaviours (McNeill et al., 2006, Yi et al., 2016). In particular, neighbourhood cohesion – referring to a sense of belonging in one’s neighbourhood and social connections shared with one’s neighbours (Buckner et al., 1988) may influence a number of health behaviours, including physical activity (McNeill et al., 2006, Yi et al., 2016).

Regularly meeting physical activity guidelines is important for health, this has been associated with reduced all-cause mortality and contributes to prevention of many chronic illnesses such as cardiovascular disease, hypertension, type 2 diabetes, obesity, osteoporosis, some cancers (e.g. breast, colon), anxiety & depression and can promote healthy cognitive function and healthy aging (Nazzari et al., 2016, Reiner et al., 2013). There is evidence that physical activity levels declined substantially as a result of pandemic restrictions (Tison, 2020) although there are few longitudinal studies that include a true physical activity pre-pandemic baseline. A reduction in physical activity has health implications, putting long term health at risk, making it a public health priority.

Previous studies have demonstrated a positive association between social support and physical activity on an interpersonal (person-to-person) level (Quinn et al., 2019) both prior to COVID-19 (Lindsay Smith et al., 2017, Scarapicchia et al., 2017) and during COVID-19 (Hailey et al., 2022). There is a small body of literature prior to the COVID-19 pandemic at community level, for example, on the relationship between social cohesion and physical activity (Echeverría et al., 2008, Murillo et al., 2016, Quinn et al., 2019, Samuel et al., 2015). This is predominantly from the USA and few employed representative population samples. A study of 23,006 respondents of the USA National Health Survey (2017), showed a positive relationship between neighbourhood cohesion and meeting physical activity guidelines. Those with higher social cohesion undertook more physical activity compared to low social cohesion, taking an extra 45 min of aerobic activity per week, and had increased odds of meeting physical activity guidance (OR = 1.14, p < 0.01) (Quinn et al., 2019). A retrospective study of changes in physical activity in 449 adults in the USA demonstrated a relationship between physical activity and social cohesion. Following a move to a ‘walkable’ community there was an increase in physical activity, social interactions, and neighbourhood cohesion reported across the sample (Zhu et al., 2014). A cross-sectional study of 2,590 Native Hawaiian and Pacific Islanders from the Native Hawaiian and Pacific Islander National Health Interview Survey (2014), compared physical activity from those in low social cohesion neighbourhoods to participants in high social cohesion neighbourhoods. This study found that high social cohesion was associated with increased odds (1.59, 95% CI: 1.19–2.12; p = 0.003) of achieving sufficient physical activity (Wang et al., 2022). These studies highlight the importance enhancing social cohesion as a potential strategy to promote physical activity. Whether these finding remain consistent, or indeed the influence of neighbourhood cohesion is even stronger, during the COVID-19 pandemic has not been examined.

The mechanism by which neighbourhood cohesion may influence physical activity is not fully understood. According to social cognitive theory, individuals with high self-efficacy are healthier and more engaged in healthy behaviours (Brand & Cheval, 2019). High self-efficacy can be developed through a strong support network, higher levels of neighbourhood cohesion may provide a strong social network, therefore an individual will be more likely to engage in health behaviours such as physical activity (Bot et al., 2016, Rosenblatt et al., 2021). Another theory is that neighbourhood cohesion increases healthy behaviours such as physical activity, by increasing awareness of chronic disease and dissemination of health-related information (Chen et al., 2019, Rosenblatt et al., 2021). Personal knowledge and awareness of disease development and prevention may be enhanced by strong social cohesion, leading to increased engagement in healthy behaviours and attendance at community level preventative healthcare initiatives (Rosenblatt et al., 2021). Another pathway is the link between social cohesion and walking through perceived walkability. Social cohesion has been shown to be positively associated with time spent walking for leisure and transport, mediated by perceived neighbourhood walkability (Koohsari et al., 2023). Perceived neighbourhood walkability has a positive effect on social cohesion via neighbourhood-based social interaction (van den Berg et al., 2022). Social cohesion may enhance perceived walkability via social interaction, positively influencing walking behaviour.

According to socio-ecological theory, several factors at the individual, social & physical environment impact an individual’s physical activity behaviour (Trost et al., 2002). Physical environmental factors, such as crime, traffic, or lack of green space may influence an individual’s ability to participate in physical activity (He et al., 2020). The social environment, such as neighbourhood cohesion and social capital, are core social environmental factors that influence health related behaviours (He et al., 2020, McNeill et al., 2006).

Factors that are associated with lower neighbourhood cohesion include poor physical and/or mental health and socio-economic insecurity (Lim & Laurence, 2015). Poor health can be associated with social isolation and economic insecurity can lead to prioritisation of resources to the immediate household rather than the community (Borkowska & Laurence, 2021). Socially and economically disadvantaged communities may also be more vulnerable due to community-level factors, such as lower social resources, cultural norms of trust and engagement or weaker civic organisation infrastructure. These may be weaker and less resilient to begin with and are important for cohesion (Lim & Laurence, 2015).

The social environment, such as neighbourhood cohesion, influence health related behaviours. Whilst there is evidence that neighbourhood cohesion had a positive effect on physical activity prior to COVID-19, this study aimed to understand if high neighbourhood cohesion continued to have a positive effect on physical activity during the pandemic as this has implications for health improvement initiatives. To the best of our knowledge this is the first study to explore these associations under pandemic restrictions and with a large sample size drawn from a representative sample of the population.

The hypothesis of this study was that overall physical activity dropped through the COVID-19 pandemic and that higher neighbourhood cohesion was a protective factor against reduced physical activity during the pandemic. Therefore, the aim was to examine the association between neighbourhood cohesion and physical activity trajectories during the COVID-19 pandemic using nationally representative longitudinal data of respondents aged 16 years and older from the UK-based *Understanding Society* COVID-19 sub-study.

## 2 Methods

### 2.1 Participants

Participants were drawn from *Understanding Society*: the UK Household Longitudinal Study (UKHLS) COVID-19 sub-study. *Understanding Society* is a representative longitudinal study of 40,000 British households followed since 2009 (Buck & Mcfall, 2012). Respondents aged 16 years and over completed the adult survey. Data were collected by trained interviewers every 2 years via face-to-face interviews (Buck and Mcfall, 2012, University of Essex, 2022). The *Understanding Society* COVID-19 sub-study intended to capture individuals’ experiences during the pandemic. This was conducted via repeat online surveys. Those in households who had participated in at least one of the previous two waves (wave 8 or 9) of data collection and were aged ≥16 years (as of April 2020) were eligible for the COVID-19 Study. Those who refused, or who were mentally or physically unable to make an informed decision to take part and those with an unknown or address abroad were excluded (Institute for Social and Economic Research, 2021). As the COVID-19 Study sample consists of active members of the main study, this allows data to be linked to information provided in pre-COVID-19 waves.

Participants completed a regular web-based survey which included core content, designed to track change over time, and variable content modules. COVID-19 data collection started April 2020, and finished in September 2021. This paper utilises neighbourhood cohesion from main survey wave 9 (Jan 2017–Dec 2019) and physical activity data from the main survey, wave 9 (Jan 2017–Dec 2019), and COVID-19, waves 1 (April 2020), 5 (September 2020) and 7 (January 2021). Physical activity was a rotating module topic rather than a repeated measure and was assessed in these waves. Participants were included in the current study if they had baseline data (physical activity and neighbourhood cohesion) and at least one measure of physical activity from one of the three COVID-19 waves. *Understanding Society*: the UK Household Longitudinal Study is a publicly available, anonymised, dataset, and thus exempt from ethical compliance and UCL Ethics Review.

## 3 Measures

### 3.1 Dependent variable – *Physical activity*

Physical activity was assessed using the International Physical Activity Questionnaire (IPAQ) short form, an internationally used instrument of self-report physical activity. IPAQ comprises 4 questions, aiming to measure the volume and intensity of physical activity performed over the last 7 days. The questions ask about the duration (days per week and minutes per day), and intensity of physical activity (vigorous, moderate, and walking). Sedentary (sitting) behaviour data was not collected. IPAQ is shown to be reliable and at least as valid as other physical activity measures for adults aged 18–65 (Craig et al., 2003) and validated for use with older adults (≥65 years) (Cleland et al., 2018, Tomioka et al., 2011).

Participant response to IPAQ were used to estimate the total amount of physical activity completed over a seven-day period in metabolic equivalents (MET). For each category the duration (hours & minutes) and frequency (days) were used to calculate the total number of minutes of activity for each category across a 7-day period. This was multiplied by the weighted MET estimate for each category and added together to produce a total physical activity per week (MET-min/wk). See Supplemental Table 1 for the weighted estimate for each category and associated calculation.

Physical activity data was processed and analysed following the current IPAQ data usage guidelines (IPAQ, 2005). If participants reported implausible physical activity levels, the observation was excluded from analysis. This included any participant who reported a total activity time > 960 min (16 h) per day, assuming an average of 16 h of waking time. Those who reported < 10 min of activity per day were recoded to zero. Finally, data were truncated as in previous studies so that individuals exceeding 180 min in any intensity category were recoded as 180 min, permitting a maximum of 21 h of activity in a week for each category (IPAQ, 2005). Data processing guidelines were followed to maintain the reliability and validity of the questionnaire and allow for comparison with other studies that used IPAQ data.

For descriptive purposes physical activity was categorised into three levels of activity. ‘Low activity’ (1–449 MET-min/week) defined as not meeting any criteria of physical activity. ‘Moderate activity’ (450–894 MET-min/week) equivalent to ‘half an hour of at least moderate-intensity PA on most days’, and therefore likely to be meeting current PA guidelines of 150 min of moderate intensity physical activity (MVPA) per week (Bull et al., 2020). ‘High activity’ (895–1794 MET-min/week) describes highly active participants which equates to ≥1 h per day, of at least moderate-intensity activity (IPAQ, 2005). (Bull et al., 2020) A clinically meaningful shift in MET-min/wk is hard to quantify. Current World Health Organisation guidelines recommend replacing sedentary behaviour with any intensity of activity for health benefits, doing some physical activity is better than doing none (Bull et al., 2020). A large systematic review of 196 articles suggested that the greatest population health benefits can be achieved by getting inactive people undertaking small increases in physical activity (Garcia et al., 2023).

## 4 Independent variable – social environment

### 4.1 Neighbourhood cohesion

Neighbourhood cohesion was assessed at the baseline of the present study, main survey wave 9 (Jan 2017–Dec 2019), using Buckner’s Neighbourhood Cohesion Instrument. This instrument was developed incorporating three key domains; the concepts of psychological sense of community, attraction to the neighbourhood, and social interaction within the neighbourhood (Buckner et al., 1988). For *Understanding Society* the original 18-question instrument was adapted into an eight-question scale (McCulloch, 2003), which has been previously validated (University of Essex, 2022). The questions are measured on a 5-point Likert scale (1- strongly agree; 5-strongly disagree), computed as the mean reverse coded response to the original variables. Higher values represent greater cohesion, ranging from 1 “lowest cohesion” to 5 “highest cohesion”. Example question, ‘I can borrow things from neighbours’ See Supplemental Table 2 for the eight-questions in Buckner’s neighbourhood cohesion score.

## 5 Covariates

Socioeconomic covariates included sex (male or female), age in years, ethnicity (white or non-white), employment status (employed, unemployed, student, retired), higher education (yes or no), and urbanicity (urban or rural). Other covariates included long standing illness or disability (yes or no).

## 6 Analysis

### 6.1 Statistical analysis

We used linear mixed models with a random intercept and slope at the individual level and an unstructured correlation matrix to examine the association between neighbourhood cohesion and physical activity during the follow-up period. These models accommodate missing data, allowing us to use all available data over the follow-up, and account for intra-individual clustering. Interactions with age were checked and were not significant, models were centred at age 50 and were adjusted for all covariates and their interactions with age at baseline, significant based on the Wald test. There was not a significant time period (39 months) when considering someone’s difference such as sex, education status etc. Analysis was carried out using Stata 17.0 (StataCorp, College Station, TX) statistical software with a two-sided p-value < 0.05 considered to be significant. Continuous variables for physical activity (MET-min/wk) and neighbourhood cohesion (Buckner’s Neighbourhood Cohesion Instrument) were used in the main analysis, categorical physical activity data were used for descriptive purposes to describe the proportion of people likely meeting/not meeting current physical activity guidelines. We checked if neighbourhood cohesion would fit categorically however Buckner’s Neighbourhood Cohesion Instrument cannot fit categorically as it is not an integer scale. Higher order interactions were checked and were found to be non-significant.

## 7 Results

### 7.1 Sample characteristics

28,268 participants had complete baseline data, listwise deletion was utilised to manage missing data prior to analysis. Those with complete baseline data and at least one Covid physical activity measure were included in the current study, N = 14,475. See supplemental Fig. 1 for flowchart of study participant selection. They were predominantly female (58%), white (British/other) (88%), mean age was 50 years (SD = 16.3) range 16–95 years, two thirds reporting being employed, with almost half having achieved higher education. Three quarters reported living in an urban setting and a third reported having a long-standing health condition. There are few differences between baseline and study participants, the study population has a slightly higher number of white, higher educated and working people than baseline (Table 1). In wave 9 of them main survey, participants were asked if they planned to move home in 2017–2019 (prior to the next survey), 3.4% of participants reported they were considering moving. Due to the low percentage, it was thought unlikely to impact the outcome of this study.

**Table 1:** Description of sample characteristics of study and baseline participants from *Understand Society Study* (wave 9, Jan 2017-Dec 2019), and COVID-19 sub-study (waves 1, 5, 7).

	**Study participants**		**Baseline** **Wave 9 (17/18)**	
**Variable**	**Number** (N = 14,475)	**%**	**Number** (N **=** 28,268)	**%**
**Sex** MaleFemale	8,426	58.2	15,710	55.6
**Ethnicity**White (British/Other) BAME	1,817	**87.5**12.5	4,947	17.5
**Employment status** Employed Unemployed StudentRetired	1,283 7333,206	**63.9** 8.9 5.122.1	3,254 1,9606,478	11.5 6.922.9
**Higher Education**Yes		**47.4**		
**Urban living**Yes				
**Long-standing health condition**Yes	4,854	33.5	9,880	35.0

### 7.2 Neighbourhood cohesion and physical activity

**Fig. 1:**
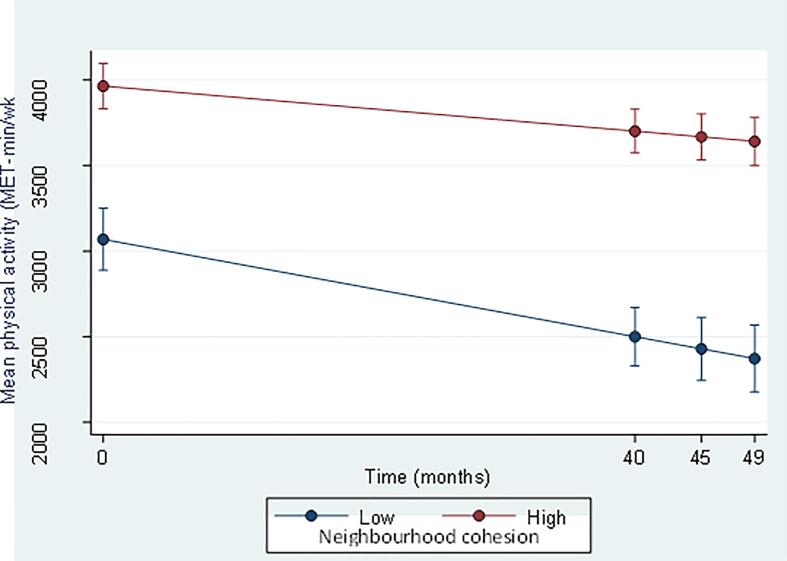
Mean physical activity (MET-min/wk) over time (49 months, COVID-19 sub-study (waves 1, 5, 7) by baseline neighbourhood cohesion (*Understand Society Study* wave 9, 2017–2019).

At baseline, the mean neighbourhood cohesion score was 3.5 (SE 0.003) and mean physical activity was 2934 MET-min/wk (SE 25.29). There was an average reduction of −441 MET-min/wk, (CI 374.51 – 507.65, p < 0.001) from baseline to the end of the follow up period. At baseline there was an association between neighbourhood cohesion and physical activity, after adjusting for sex, age, ethnicity, employment status, higher education, urban living and long-term health condition, with a one unit increase in neighbourhood cohesion corresponding to an increase in physical activity of 193 MET-min/wk (95% CI 39.88 – 346.80, p = 0.014). The difference in physical activity at baseline between the highest and lowest neighbourhood cohesion was 896 MET-min/wk (95% CI 639.9 – 1151.6, p < 0.001), and at the end of follow up it was 1269 MET-min/wk (95% CI 989.3 – 1549.1, p = 0.000), (Fig. 1). There was a significant difference between neighbourhood cohesion categories and change seen in physical activity during the 39-month follow-up period. The difference in change between lowest and highest neighbourhood cohesion categories was 373 MET-min/wk, p = 0.036; see Fig. 1. This supports the hypothesis that higher neighbourhood cohesion was protective to reduced physical activity during the pandemic.

The percentage reporting being inactive increased from 24.1% to 32.8%, see Supplemental Table 3 for full details. The biggest change was seen in those who reported being highly active (participants reporting to ≥1 h per day of at least moderate-intensity activity), dropping from 41.3% to 31.8%. Those who reported being active most days remained fairly stable.

## 8 Discussion

This study aimed to examine associations between neighbourhood cohesion and physical activity trajectories during COVID-19. The findings support our hypothesis, the COVID-19 pandemic had an overall negative impact on physical activity with a significant reduction in physical activity reported (-441 MET-min/wk, p < 0.001). And higher neighbourhood cohesion was related to higher physical activity, these differences were maintained through the pandemic with a slower decline in physical activity over time for participants with higher neighbourhood cohesion suggesting it has a protective effect on activity.

As hybrid working continues as the new normal, neighbourhood cohesion may become more important to physical activity levels as people spend more time in their neighbourhood both working and living. An increase in facilities will be needed to support physical activity both in the office working environment and for those working from home, so people can be active in both environments.

In this study we identified the pandemic led to an overall reduction in physical activity, as the pandemic recedes activity levels are starting to recovery however they haven’t returned to pre-pandemic levels. This has long term implications for health and wellbeing beyond the pandemic and requires action to reinvigorate and support public health initiatives to increase physical activity. Supporting efforts to re-engage people into physical activity through enabling social cohesion or promoting group schemes/activities is encouraged. This study highlights the importance of neighbourhood cohesion and suggest it could be a protective factor against reduced physical activity during and potentially beyond the pandemic, an area for future research.

Studies from previous environmental disasters suggest that higher perceived neighbourhood cohesion creates greater community resilience which aids in faster recovery from natural disaster (Cagney et al., 2016), individuals living in more cohesive neighbourhoods were more likely to be positive about the future (Jung, 2019). A study exploring the impact of the COVID-19 pandemic on neighbourhood cohesion showed that levels in June 2020 were lower compared to the pre-pandemic period. They also noted that the decline was particularly high in vulnerable groups (deprived communities, some ethnic minority groups and lower-skilled workers) (Borkowska & Laurence, 2021).

Our study showed that people with lower neighbourhood cohesion undertook less physical activity. Individuals from vulnerable groups and with lower cohesion are at particular risk of low physical activity with the associated risk to physical and mental health. Including a ‘social cohesion assessment’ as part of a health and wellbeing review could help identify those at increased risk of low physical activity due to low social cohesion and promote social or group interventions in order to support these already vulnerable groups.

The strengths of this study include a true pre-COVID-19 baseline, with data captured from January 2017 to December 2019, making it close to the period of interest but unaffected by the COVID-19 pandemic. Few studies have true baseline physical activity data, most rely on reporting activity levels in the weeks prior to the pandemic leading to recall bias and physical activity that may have already been affected by the pandemic. There were multiple waves of physical activity data collected which allowed for longitudinal analysis. Validated scales were used to capture physical activity and neighbourhood cohesion, these scales provide reliable output which can be compared to other studies using the same scale. A large sample size increases the reliability and generalisability of the results. Limitations of the study are that while *Understanding Society* is representative of the UK population, the Covid subsample was not thereby reducing generalisability of the results to the general population. The study sample had a slightly higher proportion of white, employed and higher educated participants than the baseline study population, sex, urban living and having a health condition stayed the same. Our study captures data to mid-way through the pandemic, there could be further long-term effect on physical activity which have not been captured. The last time point was January 2021, there is a known drop in physical activity seen during the winter months (Turrisi et al., 2021), the drop seen here could be related to seasonality rather than the pandemic. Self-reported physical activity was used in this study, potentially reducing the validity and reliability of the measure due to recall bias, a validated scale was used to help mitigate this. A further limitation was that the neighbourhood cohesion data was collected prior to the pandemic, restrictions put in place such as, lockdown and social distancing may have changed neighbourhood cohesion during this time with subsequent impact on physical activity.

Future studies should evaluate the effectiveness of policies aimed at improving neighbourhood social environment on physical activity and subsequently the long-term health and well-being benefits within neighbourhoods. Current policies aim to improve the overall neighbourhood social environment, realistic time scales for this to occur and the subsequent impact on physical activity and health is essential for effective evaluation. Identifying if overall neighbourhood cohesion or a sub-domain of neighbourhood cohesion affects physical activity would allow for targeted interventions. Additionally, different levels of activity may have different requirements, looking at components of neighbourhood cohesion with different levels of activity could allow for more strategic interventions.

## 9 Conclusion

Findings support that the COVID-19 pandemic had an overall negative impact on physical activity. Results demonstrated that high neighbourhood cohesion has a positive associated with physical activity, and this continued throughout the pandemic. As hybrid working continues as the new normal, neighbourhood cohesion may become more important as people spend more time in their neighbourhood both working and living. An increase in facilities will be needed to support physical activity in both the office working environment and for those working from home. Building communities with structures in place to develop a sense of community, allow social interaction and attraction to the neighbourhood should be a priority to help build long-term neighbourhood cohesion and subsequently physical activity. Strong relationships between public health and urban planning and design sectors are needed to develop and support local strategies in response to the health challenges that have been identified during the pandemic.

## Disclosure of funding

VH was funded by the ESRC-BBSRC Soc-B Centre for Doctoral Training, ES/P000347/1.

## Declaration of Competing Interest

The authors declare that they have no known competing financial interests or personal relationships that could have appeared to influence the work reported in this paper.

## Data Availability

Data will be made available on request.
