# Supplementary material for: Association between neighbourhood cohesion and physical activity trajectories during the COVID-19 pandemic using data from Understanding Society: The UK Household Longitudinal Study & COVID-19 sub-study
Source: Prev Med Rep. 2023 Aug 29;35:102392. doi: 10.1016/j.pmedr.2023.102392 (PMC10480663; doi:10.1016/j.pmedr.2023.102392)
Supplement: Supplementary data 1 [file mmc1.docx]

**Supplemental Table 1. Calculation of MET-min/wk variables for IPAQ to calculate a continuous score**

| **Category** | **Weighted Met level** | **Calculation of MET-min/wk** |
| --- | --- | --- |
| Walking | 3.3 METs | 3.3 x walking mins/wk |
| Moderate | 4.0 METs | 4.0 x moderate mins/wk |
| Vigorous | 8.0 METS | 8.0 x vigorous mins/wk |
| **TOTAL**  MET-min/wk |  | walking MET-min/wk + moderate MET-min/wk + vigorous MET-min/wk |

**Supplemental Table 2. Questions for Buckner’s neighbourhood cohesion**

| **#** | **Buckner’s neighbourhood cohesion index Questions** | **Concept** |
| --- | --- | --- |
| 1 | I plan to stay in the neighbourhood | Attraction to neighbourhood |
| 2 | I can borrow things from my neighbours | Measure neighbouring |
| 3 | If I needed advice, advice is obtainable locally | Measure neighbouring |
| 4 | I talk regularly with neighbours | Measure neighbouring |
| 5 | I am similar to others in this neighbourhood | Psychological sense of community |
| 6 | I feel like I belong to this neighbourhood | Psychological sense of community |
| 7 | Local friendships mean a lot to me | Psychological sense of community |
| 8 | I would be willing to improve my neighbourhood | Psychological sense of community |

**Supplemental Table 3. Categorised physical activity of *Understand Society* Study participants at four time points. Wave 9 (2017- 2019), and COVID-19 sub-study (waves 1, 5, 7)**

| **Wave** | **Total number of observations** | **Low PA** | **Medium PA** | **High PA** |
| --- | --- | --- | --- | --- |
| **2017/2018**  **(baseline)** | 14,476 | 3,492  (24.1%) | 4,999  (34.5%) | 5,985  (41.3%) |
| **April 2020**  **(Wave 1)** | 13,785 | 3,979  (28.8%) | 3,912  (28.4%) | 5,897  (42.8%) |
| **Sept 2020**  **(Wave 5)** | 10,217 | 2,943  (28.8%) | 3,303  (32.3%) | 3,971  (38.9%) |
| **Jan 2021**  **(Wave 7)** | 9,557 | 3,135  (32.8%) | 3,387  (35.4%) | 3,035  (31.8%) |

**Supplemental figure 1. Flowchart of study participant selection** from *Understand Society Study* (wave 9, 2017- 2019), and COVID-19 sub-study (waves 1, 5, 7)

Total participants wave 9

N = 35,733

Missing baseline neighbourhood cohesion or physical activity

N = 4,543

Missing baseline covariate

N = 2,922

Baseline participants

N = 28,268

No COVID-19 physical activity data

N = 13,793

Study participants

N = 14,475
